# Spatial–temporal distribution characteristics of pulmonary tuberculosis in eastern China from 2011 to 2021

**DOI:** 10.1017/S0950268824000785

**Published:** 2024-05-15

**Authors:** Ke Chen, Liang Cheng, Hao Yu, Yong Zhou, Limei Zhu, Zhongqi Li, Tenglong Li, Leonardo Martinez, Qiao Liu, Bei Wang

**Affiliations:** 1 Key Laboratory of Environmental Medicine Engineering of Ministry of Education, Department of Epidemiology and Health Statistics, School of Public Health, Southeast University, Nanjing, China; 2 Department of Tuberculosis, Affiliated Wuxi Fifth Hospital of Jiangnan University, Wuxi, China; 3 Department of Chronic Communicable Disease, Center for Disease Control and Prevention of Jiangsu Province, Nanjing, China; 4 Department of Chronic Disease, Center for Disease Control and Prevention of Heilongjiang Province, Harbin, China; 5 Academy of Pharmacy, Xi’an Jiaotong-Liverpool University, Suzhou, China; 6 Department of Epidemiology, School of Public Health, Boston University, Boston, MA, USA

**Keywords:** China, epidemiology, pulmonary tuberculosis, spatial autocorrelation, spatial–temporal scan

## Abstract

China is still among the 30 high-burden tuberculosis (TB) countries in the world. Few studies have described the spatial epidemiological characteristics of pulmonary TB (PTB) in Jiangsu Province. The registered incidence data of PTB patients in 95 counties of Jiangsu Province from 2011 to 2021 were collected from the Tuberculosis Management Information System. Three-dimensional spatial trends, spatial autocorrelation, and spatial–temporal scan analysis were conducted to explore the spatial clustering pattern of PTB. From 2011 to 2021, a total of 347,495 newly diagnosed PTB cases were registered. The registered incidence rate of PTB decreased from 49.78/100,000 in 2011 to 26.49/100,000 in 2021, exhibiting a steady downward trend (χ^2^ = 414.22, *P* < 0.001). The average annual registered incidence rate of PTB was higher in the central and northern regions. Moran’s I indices of the registered incidence of PTB were all >0 (*P*< 0.05) except in 2016, indicating a positive spatial correlation overall. Local autocorrelation analysis showed that ‘high–high’ clusters were mainly distributed in northern Jiangsu, and ‘low–low’ clusters were mainly concentrated in southern Jiangsu. The results of this study assist in identifying settings and locations of high TB risk and inform policy-making for PTB control and prevention.

## Introduction

China ranked third among the 30 high-burden tuberculosis (TB) countries in 2021, which was lower than the number of TB cases in Indonesia and India. The estimated number of TB patients accounted for 7.4% of the total global burden in 2021 [1]. In order to effectively curb the epidemic of TB, China has continuously introduced TB prevention and control measures in the past decade, and positive progress has been made in the prevention and control of TB [2, 3]. The incidence rate of TB in China reported by the National Health Commission in 2021 was 45 cases per 100,000 persons [4]; this is ahead of schedule of the target of 55 cases per 100,000 persons derived in the Action Plan to Stop Tuberculosis (2019–2022) formulated by the Chinese government [3]. Despite this improvement, the number of reported TB deaths still ranks second among class A and B infectious diseases in China [5].

Previous studies have found that the incidence of pulmonary TB (PTB) in different regions may be distinct due to geographical factors, climate, social economy, among others [6–8]. The National Tuberculosis Epidemiological Sampling Survey is a cross-sectional investigation conducted nationwide using scientific methods to sample representative populations, thereby obtaining nationwide TB prevalence data at a specific point in time. The Fifth National Tuberculosis Epidemiological Sampling Survey uncovered that there were obvious regional differences in PTB incidence in China. For example, the PTB incidence in rural areas was significantly higher than that in urban areas; in addition, the central and western regions have significantly higher incidence than in eastern regions. PTB incidence in the western region is highest, approximately 1.7 and 2.4 times that of the central and eastern regions [9]. In areas with low PTB rates, the PTB incidence was also affected by floating migrant populations in recent years [10]. Jiangsu Province is located on the eastern coast of China, and there is considerable heterogeneity in climate and economic development within the province. With the rapid economic development and the increase in the migrant population, PTB epidemics are frequently recorded in Jiangsu Province.

Spatial epidemiology has been widely used in infectious diseases in recent years to analyse links between disease distribution and change in different regions based on monitoring data. Studies from Iran [11] and Kenya [12] have reported spatial clustering of PTB at the national and county levels. Prior research describes spatial clusters of PTB at the national, provincial, municipal, and county levels in China [13–15]. Thus far, there is no study on the spatial–temporal analysis of PTB in Jiangsu Province. Therefore, we conducted a temporal, spatial, and spatial–temporal analysis of PTB incidence at the county level in Jiangsu Province from 2011 to 2021, to provide more useful information for policy-making.

## Methods

### Study area

Jiangsu Province is located on the eastern coast of China, in the Yangtze River Delta region, with a latitude and longitude of about 30°45 ’-35°08’ N, 116°21 ’-121°56′ E. The total area is 10,7200^2^ km. By the end of 2021, there were 95 counties in Jiangsu Province, with a permanent population of 85 million, and Gross Domestic Product (GDP) per capita ranked first in China.

### Data sources

The registered incidence data of PTB patients from 2011 to 2021 were obtained from the Tuberculosis Management Information System of Jiangsu Province [16–18]; statistical analysis was conducted based on the current addresses of cases. The data of permanent residents from 2011 to 2021 were collected from the statistical yearbooks of each city. Vector maps of counties in Jiangsu Province were downloaded from the database of the National Basic Geographic Information System.

### Data processing

We calculated the registered incidence rate of PTB in 95 counties of Jiangsu Province from 2011 to 2021. The ArcGIS 10.7 software was used to construct a geographic information database of PTB incidence rate, including the name, code, latitude, and longitude, and the registered incidence rate of PTB in each county, with the administrative division code as the matching field associated with the vector map.

### Descriptive and time-series analysis

The registered incidence rates of PTB in Jiangsu Province from 2011 to 2021 were computed and used in the three-dimensional spatial trend analysis by the ArcGIS 10.7 software. The spatial distribution map and the three-dimensional spatial trend analysis map of the annual registered incidence rate of PTB in the counties were subsequently drawn. The numbers of newly registered PTB cases were summarized by month, and the Excel 2013 software was used to draw the time-series diagram.

### Spatial autocorrelation analysis

Spatial autocorrelation analysis is often used to explore whether a certain feature of a spatial unit in a region is correlated with the feature of its neighbouring spatial unit, and it is often employed to measure the clustering and dispersion degree of a feature of a spatial unit [19]. In this study, the geographic information data of registered PTB cases in Jiangsu Province from 2011 to 2021 were imported into GeoDa 1.18.0 software for global autocorrelation analysis and local autocorrelation analysis. Moran’s I is a common index in global autocorrelation analysis and is used to quantify the overall distributional characteristics of a study area, as it represents the average aggregation degree of similar attributes in a study area. The value of Moran’s I ranges from −1 to 1. For a positive I, a larger value indicates a stronger spatial clustering pattern; for a negative I, a smaller value indicates a weaker spatial clustering pattern; and a zero I suggests there is no spatial clustering [20]. The local spatial autocorrelation analysis was used to analyse the spatial differences in PTB registration incidence rates at the county level via the cluster map of local spatial correlation indicators. There are four types of clusters, namely, ‘high–high’ clusters (high-incidence areas surrounded by high-incidence areas); ‘low–low’ clusters (low-incidence areas surrounded by low-incidence areas); ‘high-low’ clusters (high-incidence areas surrounded by low-incidence areas); and ‘low–high’ clusters (low-incidence areas surrounded by high-incidence areas) [21].

### Spatial–temporal scan analysis

The SaTScan 10.1 software was used to perform spatial–temporal scan analysis based on the Poisson distribution model. A cylindrical-shaped scanning window with a base of space and a height of time was established. The log-likelihood ratio (LLR) was constructed according to the actual and expected number of PTB cases inside and outside of the scanning window to estimate the risk of PTB in the window, and the relative risk (RR) was calculated to evaluate the risk of each cluster. The larger the LLR, the more statistically significant the difference was, and the higher the RR in this window, the more likely there were clustering areas [22, 23]. In this study, the maximum scanning time was set to 50% of the total study time, the maximum scanning space was set to 25% of the population, and the scanning interval was set to 1 year.

ArcGIS 10.7 software was used for the three-dimensional spatial trend analysis and the visualization of the results. The significance level was set as 0.05.

## Results

### Basic information

A total of 347,495 newly diagnosed PTB cases were registered in Jiangsu Province from 2011 to 2021, and the registered incidence rate decreased from 49.78/100,000 in 2011 to 26.49/100,000 in 2021, showing an annual downward trend yearly (χ2 = 414.22, *P* < 0.001). A total of 4,456 multidrug- and rifampicin-resistant TB (MDR/RR-TB) cases were registered from 2011 to 2021. The registered incidence rate of MDR/RR-TB cases increased from 0.24/100000 in 2011 to 0.62/100000 in 2021, showing an overall upward trend (χ2 = 254.95, *P* < 0.001), as shown in Table 1. The spatial distribution of the annual registered incidence rate of PTB in Jiangsu Province from 2011 to 2021 showed that the areas with high registered incidence rate of PTB were mostly in the central, north-western, and south-western regions of Jiangsu Province, while the registered incidence rate was relatively low in the south-east area. The top three of the annual registered incidence rates of PTB were Huai’an County (63.80/100,000), Gaochun County (58.11/100,000), and Xinyi County (58.09/100,000), as shown in Supplementary Figure S1 and Supplementary Figure S2. The temporal distribution of PTB cases showed seasonal fluctuations, with the peak mostly occurring from March to May each year, as shown in Supplementary Figure S3.

**Table 1.** Registration of TB cases in Jiangsu Province from 2011 to 2021

Year	Number of permanent residents at the end of the year (ten thousand people)	Number of registered TB cases	Registered TB incidence (per 100,000)	Number of registered MDR/RR-TB cases	Registered incidence of MDR/RR-TB (per 100,000)
2011	8,022.99	39,935	49.78	196	0.24
2012	8,119.81	39,781	48.99	334	0.41
2013	8,192.44	36,963	45.12	365	0.45
2014	8,281.09	36,301	43.84	308	0.37
2015	8,315.11	34,129	41.04	377	0.45
2016	8,381.47	31,412	37.48	370	0.44
2017	8,423.5	29,615	35.16	515	0.61
2018	8,446.19	27,562	32.63	456	0.54
2019	8,469.09	25,662	30.3	486	0.57
2020	8,477.26	23,605	27.85	522	0.62
2021	8,505.4	22,530	26.49	527	0.62

### Three-dimensional trend analysis

**Figure 1.**
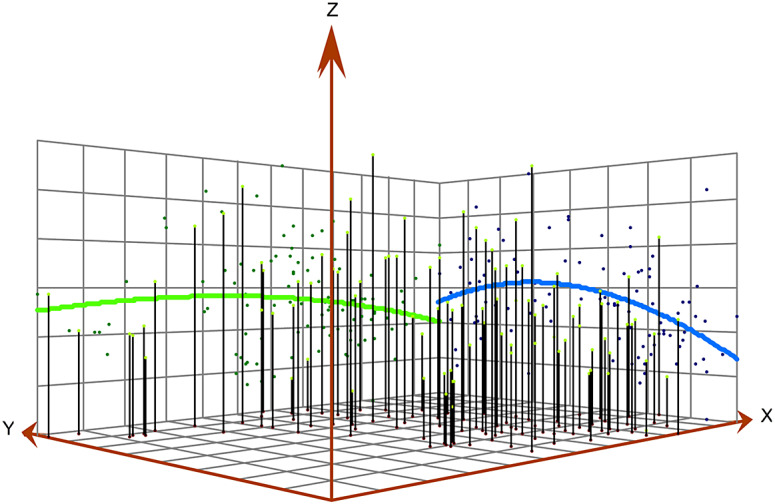
Spatial three-dimensional trend of annual registered incidence rate of TB in Jiangsu Province from 2011 to 2021 (*Z*-axis represents annual registered incidence rate, *X*-axis represents longitude, and *Y*-axis represents latitude).

The results of the three-dimensional trend map showed that the average annual registered incidence rate of PTB in Jiangsu Province from 2011 to 2021 was higher in central Jiangsu and lower in either western Jiangsu or eastern Jiangsu and showed a slow rise and then a downward trend from north to south, as shown in Figure 1.

### Global spatial autocorrelation analysis

Moran’s I values of PTB registered incidence rate in Jiangsu Province were all higher than zero in each year, and the *P* values were all lower than 0.05 during the study period in other years except for 2016, indicating that there was a positive spatial correlation and a spatial clustering distribution in PTB registered incidence rate in Jiangsu Province except for 2016 (Table 2).

**Table 2.** Global autocorrelation analysis on the registered incidence rate of TB in Jiangsu Province from 2011 to 2021

Year	Moran’s I	*Z*-value	*P*-value
2011	0.28	4.31	0.001
2012	0.32	4.95	0.001
2013	0.20	3.04	0.004
2014	0.17	2.66	0.006
2015	0.13	2.07	0.029
2016	0.09	1.61	0.061
2017	0.20	3.19	0.002
2018	0.13	2.25	0.021
2019	0.24	3.88	0.001
2020	0.24	3.69	0.001
2021	0.15	2.33	0.009
Average	0.28	4.30	0.001

### Local spatial autocorrelation analysis

**Figure 2.**
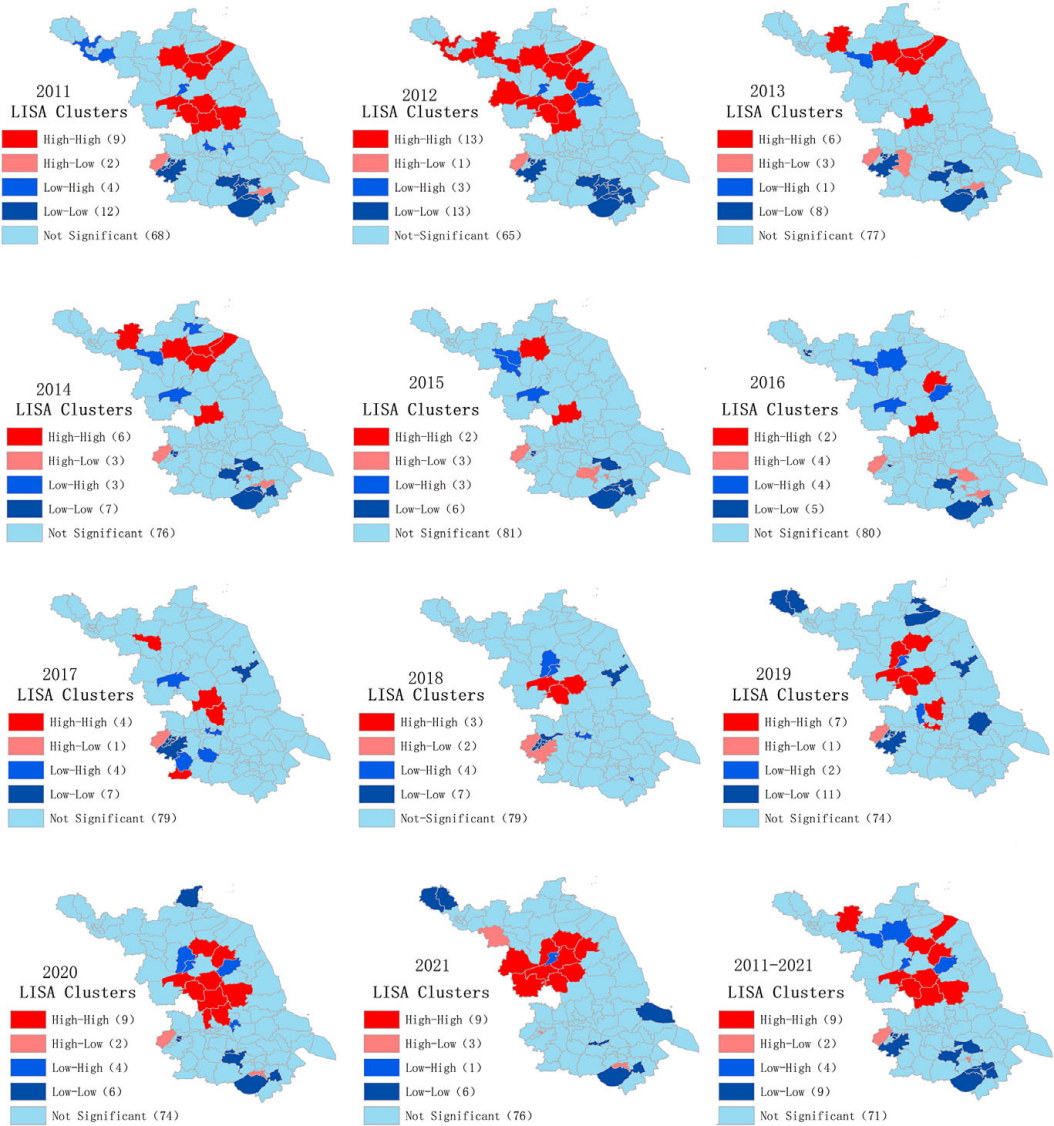
Annual LISA cluster map of registered incidence rates of TB in Jiangsu Province from 2011 to 2021.

The distribution pattern of registered incidence of PTB in Jiangsu Province was uneven. From 2011 to 2021, the ‘high–high’ clustering areas were mainly located in the northern part of Jiangsu Province, especially Huai’an, Lianyungang, and Suqian cities. The number of ‘high–high’ clustering areas was the largest in 2012, involving 13 counties. The number of ‘high–high’ clustering areas showed a downward trend from 2014 to 2018 and began to increase after 2019. The ‘low–low’ clustering areas were relatively concentrated, mainly in the southern areas such as the Wuzhong and Wujiang districts of Suzhou City and the Wujin District of Changzhou City (Figure 2). There were ‘high–high’ clustering areas in the registered incidence rate of MDR/RR-TB in Jiangsu Province from 2011 to 2021, with a dynamic distribution, with the number of counties involved concentrated in 1 to 9, of which the largest number was 9 in 2021 and the smallest number was 1 in 2019. The Library and Information Science Abstracts (LISA) results of the annual registered incidence rate of MDR/RR-TB showed that there were seven ‘high–high’ clustering areas. The number of counties and districts involved in ‘low–low’ clustering areas ranged from 1 to 12, showing a dynamic distribution, of which the maximum number was 12 in 2012 and 2017, and the minimum number was 1 in 2015, as shown in Supplementary Figure S4.

### Spatial–temporal scan analysis

**Figure 3.**
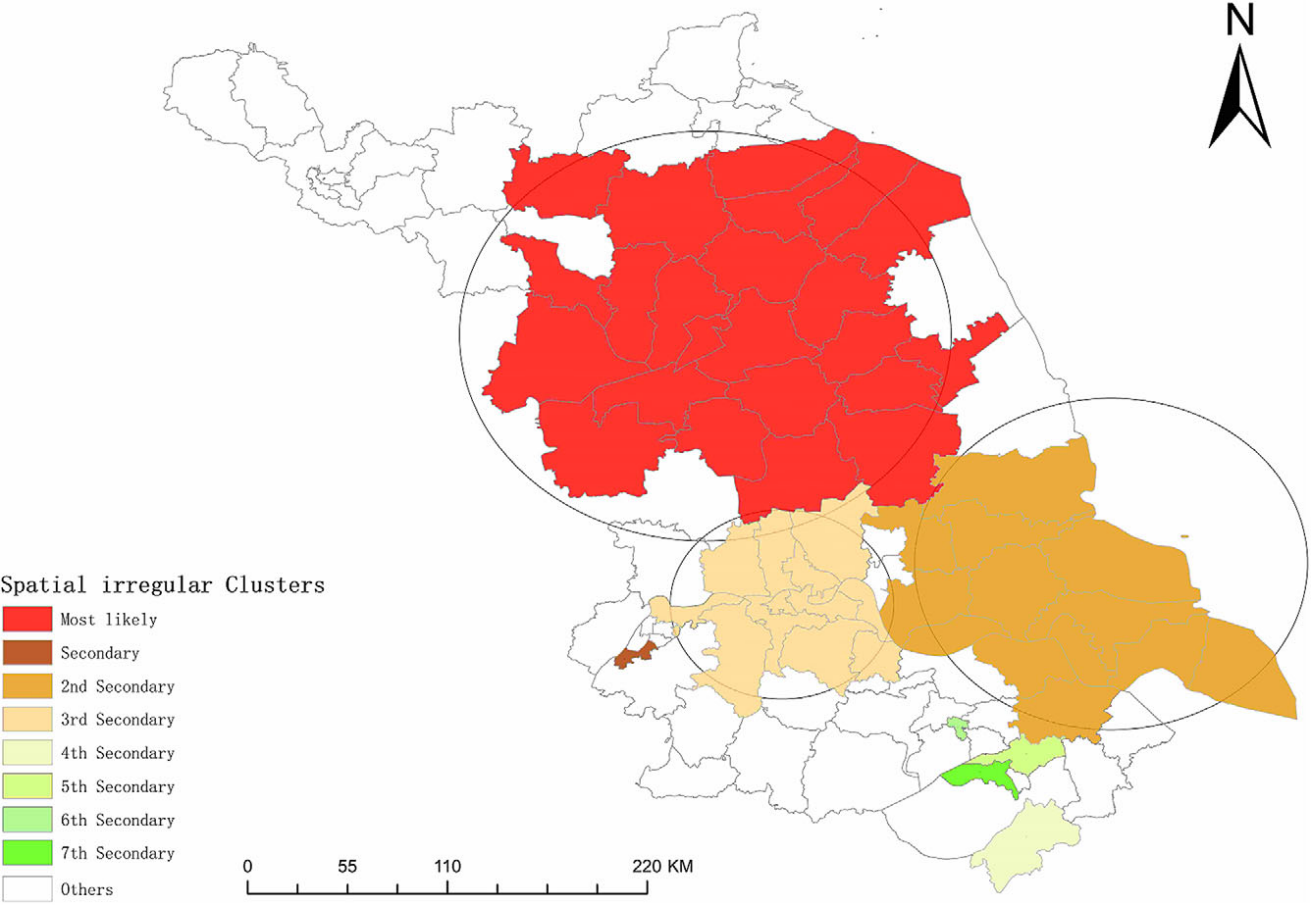
Spatial–temporal scanning characteristics of registered TB cases in Jiangsu Province from 2011 to 2021.

The results of spatial–temporal scan analysis showed that there was a spatial–temporal clustering of the registered PTB incidence rate in Jiangsu Province from 2011 to 2021, and a total of eight spatial–temporal clusters were identified (*P* < 0.05). The cluster with the highest confidence covered 23 counties, including all counties of Huai’an City, Tinghu County, Yandu County, Xiangshui County, Binhai County, Funing County, and Jianhu County of Yancheng City. Sucheng County, Shuyang County, Siyang County, and Sihong County of Suqian City, Guannan County and Guanyun County of Lianyungang City, Baoying County and Gaoyou County of Yangzhou City, Xinyi City of Xuzhou City, and Xinghua City of Taizhou City were all gathered from 2011 to 2015 (Table 3, Figure 3, and Supplementary Table S1).

**Table 3.** Spatial–temporal scan analysis of registered TB cases in Jiangsu Province from 2011 to 2021

Cluster type	Cluster time	Counties (*n*)	Radius(km)	Observed cases (*n*)	Excepted cases (*n*)	RR	LLR	*P*-value
Most likely	2011–2015	23	112.83	52,085	36,904.10	1.48	3,137.82	0.001
Secondary	2011–2015	1	0	2,411	830.05	2.92	993.49	0.001
2nd secondary	2011–2015	13	91.35	32,752	26,691.71	1.25	698.06	0.001
3rd secondary	2011–2015	12	52.07	18,942	14,584.47	1.32	622.69	0.001
4th secondary	2011–2014	1	0	2,445	2024.43	1.21	41.19	0.001
5th secondary	2011–2014	1	0	1,383	1,124.15	1.23	27.84	0.001
6th secondary	2013–2015	1	0	1,349	1,120.46	1.20	21.94	0.001
7th secondary	2018	1	0	320	235.15	1.36	13.75	0.001

## Discussion

Based on this geographic information system and spatial analysis, this study characterizes the spatial–temporal distribution of PTB cases in Jiangsu Province. This is the first study to estimate and summarize the spatial–temporal distribution characteristics of PTB at the county level in Jiangsu Province. With the implementation of the World Health Organization’s End TB Strategy by 2035, countries around the world are increasingly focusing on TB and making continuous efforts to achieve the goal of ending it. A study of the spatiotemporal distribution of PTB can help reveal its geographical distribution, epidemic trends, and clustering patterns, thereby providing an important basis for the development of more accurate TB prevention and control strategies. Analysing the spatiotemporal clustering patterns of PTB at the county level can identify high-risk counties, which can assist health administrative departments in more effectively allocating TB control resources.

During the study period, the registered PTB incidence rate in Jiangsu Province decreased from 50 cases to 26.49 cases per 100,000 persons from 2011 to 2021. A downward trend by year was seen, consistent with the national trend of PTB incidence rate during the same period. The overall incidence rate in Jiangsu Province was significantly lower than the national average [24], indicating the high effectiveness of PTB prevention and control in Jiangsu, perhaps due to the province’s increased attention to PTB prevention and control. The Jiangsu Provincial government released the 12th Five-Year Plan for Tuberculosis Prevention and Control in May 2012 [25]. The plan requires that medical institutions detect patients early, use strict diagnosis and treatment standards, and improve the level of anti-TB-related treatment [26]. In the Jiangsu Provincial Tuberculosis Prevention and Control Plan (2018–2020) [27], Jiangsu took the lead in establishing a new comprehensive PTB prevention and control service model, distributing a free supply of second-line anti-TB drugs to PTB patients, and offering free screening and diagnoses for PTB patients with suspicious symptoms. In the ‘14th Five-Year Plan’ for PTB prevention and control in Jiangsu Province released in November 2021 [28], further feasible measures were taken to effectively control the epidemic of PTB and protect the health of the people. The analysis of this study reveals that the registered incidence rate of MDR/RR-TB in Jiangsu Province from 2011 to 2021 was 0.49/100,000, indicating a low detection level. However, there has been an overall upward trend in the registered incidence, suggesting an improvement in the detection of MDR/RR-TB patients. The registered incidence rate showed a significant increase after 2017, which could be attributed to the implementation of the MDR/RR-TB project in Jiangsu Province in recent years. This project has played a vital role in enhancing the treatment management mode, detection level, and professional capabilities of the prevention and control personnel in Jiangsu Province.

Our study shows that the registered PTB incidence rate in Jiangsu Province has obvious seasonal variations. The number of registered PTB cases exhibits a clear downward trend from January to February and starts to approach its peak from March to May. There are several possible explanations for this seasonal trend. First, in autumn and winter, the decrease in ultraviolet (UV) exposure from outdoor sunlight and the increase in indoor activities may increase the chance of PTB infection [29, 30]. Second, after the incubation period, the onset of PTB typically occurs from March to May. During the same time period, we also notice the high incidence of respiratory diseases in spring and the peak of seeking medical treatment after the Spring Festival [31, 32]. Third, during the Spring Festival in China (January to February), patients are less motivated to seek medical treatment as they are busy celebrating the holiday [33]. The seasonal trend observed in this study is consistent with the findings in other studies in Jiangsu Province [34], as well as previous studies in Chongqing Municipality [15] and Hubei Province [20].

We also found substantial heterogeneity in terms of the average annual registered incidence rate of PTB within counties in Jiangsu Province during the study time period. The global spatial autocorrelation analysis found a positive spatial correlation in general and overall spatial clustering distribution of registered PTB incidence rate in Jiangsu Province, suggesting that the incidence rate of PTB in Jiangsu Province is unevenly distributed at the county level. Further local spatial autocorrelation results identified some ‘high–high’ clusters – such as Huai’an City, Suqian City, and Lianyungang City – from 2011 to 2021. The distribution of these clusters, which were mainly located in northern Jiangsu, was relatively stable. The annual registered incidence rates of PTB in these counties were high, and regional transmission is likely in these areas. Targeting areas with heavy and consistent ‘high–high’ clusters may be pertinent for reducing community-level TB transmission [35]. The ‘low–low’ clusters were mainly located in southern Jiangsu, including Wuzhong County and Wujiang County of Suzhou City. Studies have shown that the PTB incidence rate is related to levels of local economic and social development, health resources, social culture, environment, and other factors. Better urban development and economic levels are important for controlling the incidence of PTB, which is supported by our findings as the economic level of northern Jiangsu is less developed than southern Jiangsu.

The spatial–temporal scan analysis identified eight spatial–temporal clusters from 2011 to 2021, concentrated during the period of 2011–2018. There were no clear spatial–temporal clusters after 2019, indicating that the PTB burden in Jiangsu has been gradually reduced and the control of PTB has progressed. The identified clusters covered 23 counties, mainly located in the central and northern parts of Jiangsu Province, such as Huai’an, Yancheng, Suqian, and Yangzhou. The clustering pattern was the strongest from 2011 to 2015. Incidence rates of the identified clusters were higher than average levels from the whole province. Ongoing TB control measures should strengthen the surveillance and management of PTB in these areas.

There were several limitations in this study. First of all, the PTB registration incidence data were collected from the Tuberculosis Management Information System, and similar to most TB registries, there may be missed diagnoses or notifications due to underreporting; this may result in underestimation of the estimated incidence. Second, relevant factors such as socioeconomic status, climatic conditions, and personal hygiene practices were not considered in this study. Third, this study was analysed at the county level, and further studies at more refined (such as townships) levels are needed.

In conclusion, the registration incidence rate of TB in Jiangsu Province has shown a downward trend from 2011 to 2021, with peaks occurring from March to May each year. In this study, we have identified significant spatiotemporal clustering patterns and regional differences. Although the burden of TB in Jiangsu Province has been alleviated in recent years, disease control agencies should pay extra attention to the prevention and control of TB in ‘high–high’ clustering areas and spatial–temporal cluster areas, potentially by increasing the special funding for PTB, enhancing the treatment and follow-up management of PTB patients, and expanding the active screening of PTB in communities.

## Supporting information

Chen et al. supplementary material Chen et al. supplementary material

## Data Availability

Please contact the first author for data requests.

## Supplementary material

For supplementary material accompanying this paper visit http://doi.org/10.1017/S0950268824000785. click here to view supplementary material
