# Supplementary material for: Spatial–temporal distribution characteristics of pulmonary tuberculosis in eastern China from 2011 to 2021
Source: Epidemiol Infect. 2024 May 15;152:e84. doi: 10.1017/S0950268824000785 (PMC11149027; doi:10.1017/S0950268824000785)
Supplement: Chen et al. supplementary material [file S0950268824000785sup001.docx]

**Supplementary Tables and Figures.**

Spatial-temporal distribution characteristics of pulmonary tuberculosis in eastern China from 2011 to 2021

Ke Chen, Liang Chen, Hao Yu, Limei Zhu, Tenglong Li, Leonardo Martinez, Qiao Liu, Bei Wang

Supplementary Table 1. High-high clustering areas in Jiangsu Province from 2011 to 2021.

Supplementary Figure 1. Registered incidence of Pulmonary tuberculosis from 2011-2021 in Jiangsu Province.

Supplementary Figure 2. Average registered incidence of Pulmonary tuberculosis from 2011-2021 in Jiangsu Province.

Supplementary Figure 3. Trend map of registered pulmonary tuberculosis patients from 2011-2021.

Supplementary Table 1. High-high clustering areas in Jiangsu Province from 2011 to 2021

| Year | Name of counties | |
| --- | --- | --- |
| 2011 | Shuyang, Baoying, Gaoyou, Xinghua, Hongze, Lianshui, Jinhu, Xiangshui, Guannan | |
| 2012 | Suyu, Shuyang,Sihong, Tongshan, Pizhou, Baoying, Gaoyou, Hongze, Lianshui, Jinhu, Xiangshui, Funing | |
| 2013 | Shuyang, Pizhou, Gaoyou, Lianshui, Xiangshui, Guannan | |
| 2014 | Shuyang, Pizhou, Gaoyou, Lianshui, Xiangshui, Guannan | |
| 2015 | Shuyang, Gaoyou | |
| 2016 | Gaoyou, Nanning | |
| 2017 | Chongchuan, Shuyang, Baoying, Xishan | |
| 2018 | Yizheng, Lianshui, Tinghu | |
| 2019 | Jiangdu, Baoying, Huaiyin, Hongze, Lianshui, Jinhu, Guannan | |
| 2020 | Hanjiang, Jiangdu, Baoying, Gaoyou, Xinghua, Hongze, Lianshui, Jinhu, Funing | |
| 2021 | Sihong, Baoying, Huaian, Huaiyin, Hongze, Lianshui, Xuyi, Jinhu, Funing |  |


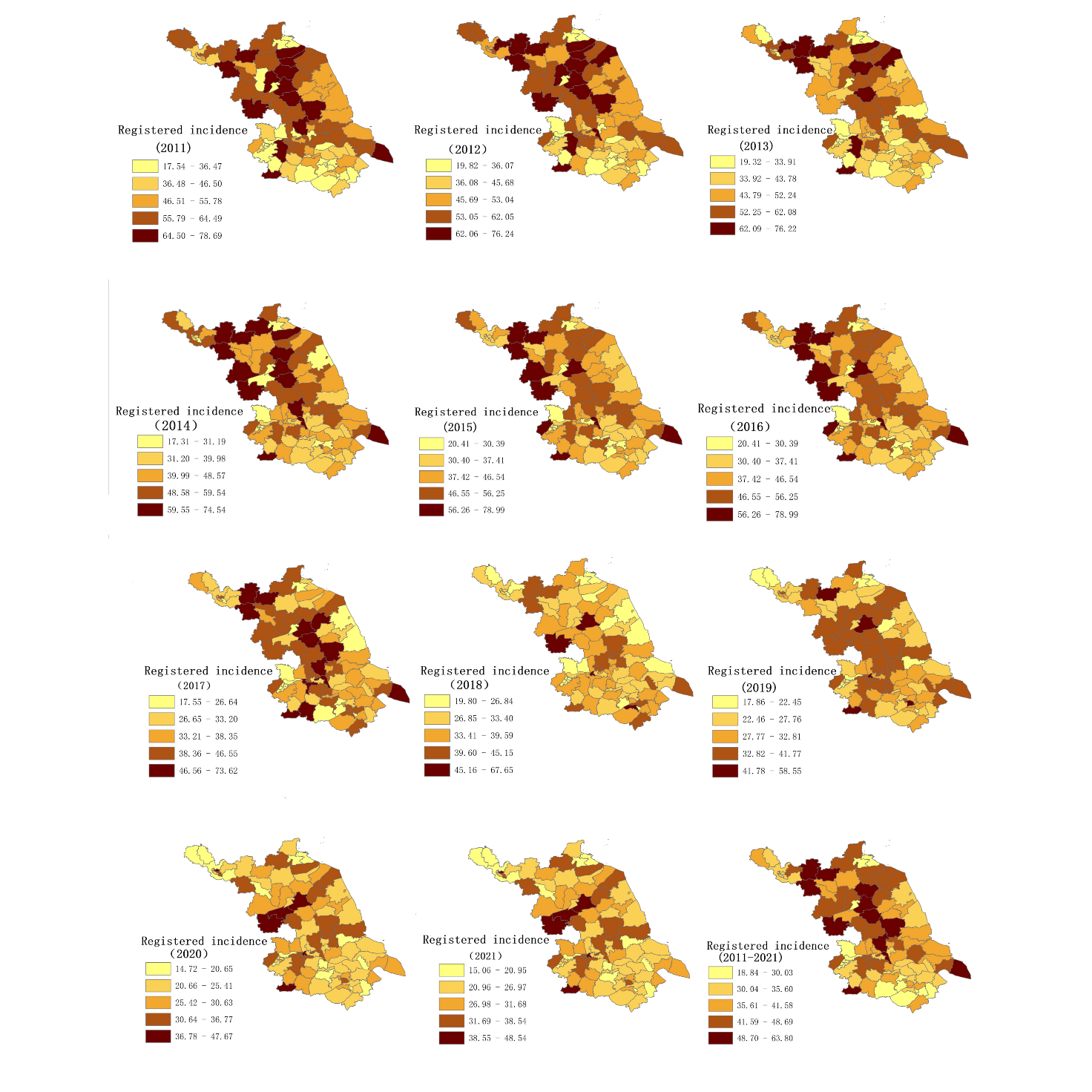


Supplementary Figure 1. Registered incidence of Pulmonary tuberculosis from 2011-2021 in Jiangsu Province.


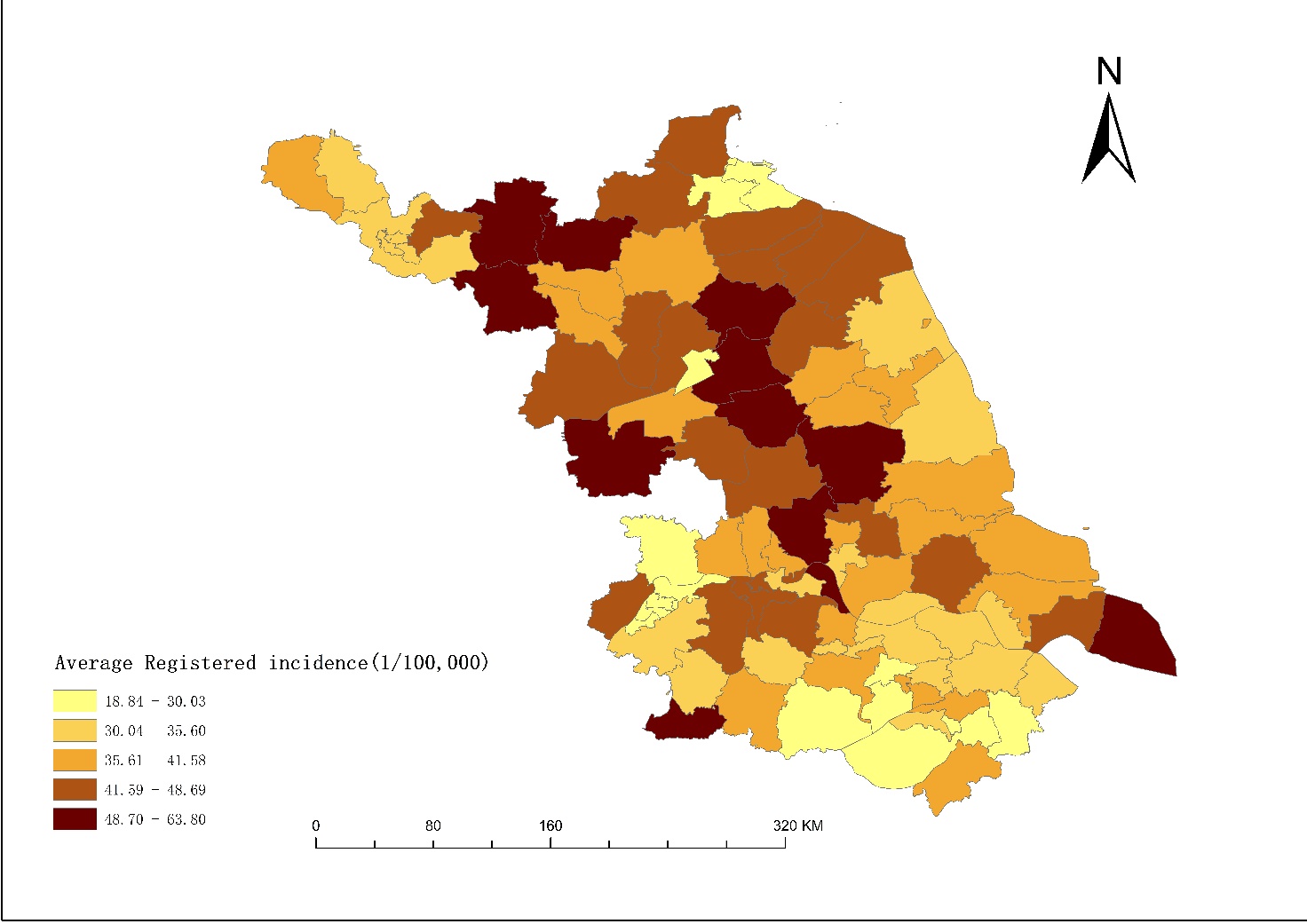


Supplementary Figure 2. Average registered incidence of Pulmonary tuberculosis from 2011-2021 in Jiangsu Province


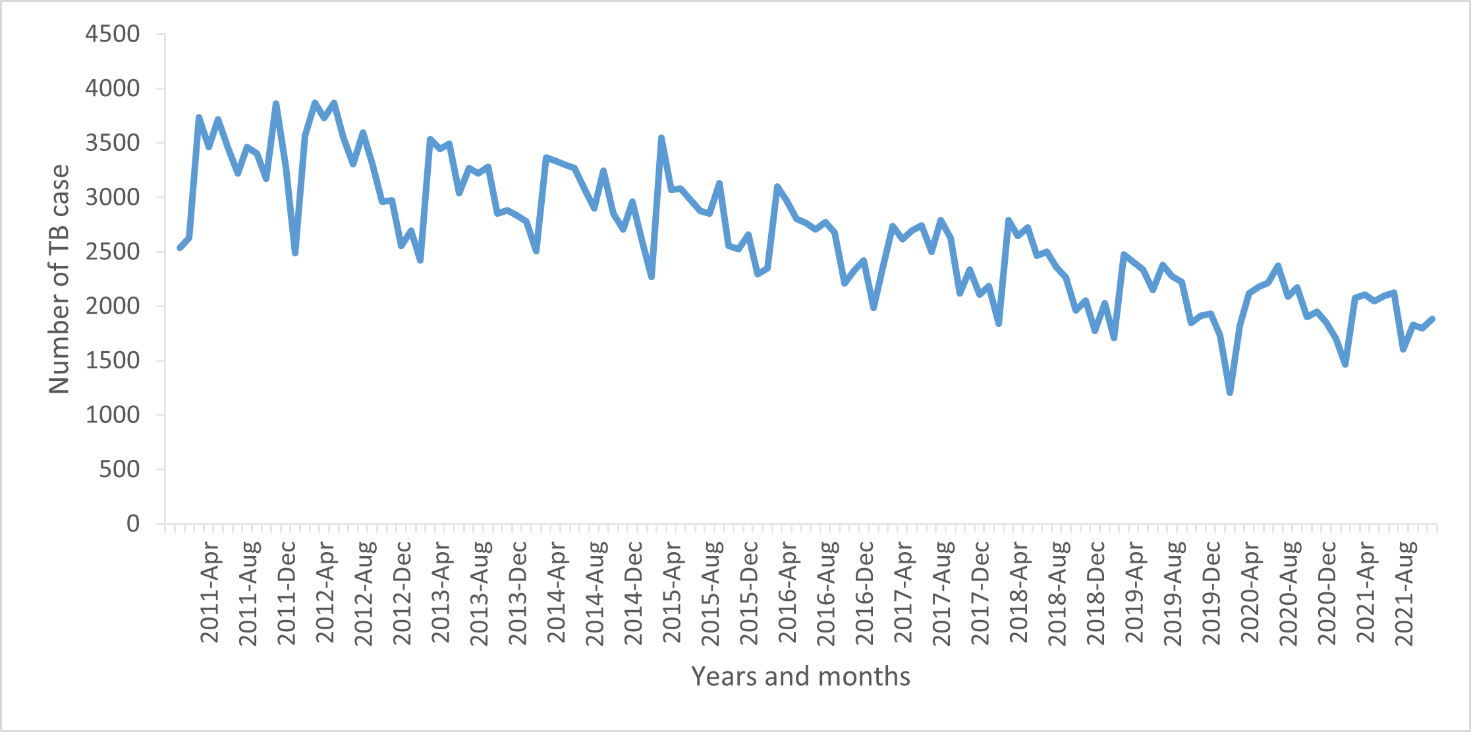


Supplementary Figure 3. Trend map of registered pulmonary tuberculosis patients from 2011-2021
